# The Impact of Mobile Health Interventions on Antenatal and Postnatal Care Utilization in Low- and Middle-Income Countries: A Meta-Analysis

**DOI:** 10.7759/cureus.21256

**Published:** 2022-01-14

**Authors:** Poonam Yadav, Ravi Kant, Surekha Kishore, Shruti Barnwal, Meenakshi Khapre

**Affiliations:** 1 College of Nursing, All India Institute of Medical Sciences, Rishikesh, IND; 2 General Medicine, All India Institute of Medical Sciences, Rishikesh, IND; 3 Community and Family Medicine, All India Institute of Medical Sciences, Gorakhpur, IND; 4 Department of Dermatology, Government Doon Medical College, Dehradun, IND; 5 Community and Family Medicine, All India Institute of Medical Sciences, Rishikesh, IND

**Keywords:** reproductive health services, maternal and child health services, maternal health, health promotion, access to health care

## Abstract

Despite many efforts, the utilization of full antenatal and postnatal care remains a significant concern in low- and middle-income countries (LMICs). We planned to retrieve the relevant literature and pooled the data for meta-analysis to evaluate the impact of mobile health (mHealth) intervention on antenatal and postnatal care utilization in low- and middle-income countries. We searched the literature through major electronic databases such as PubMed, MEDLINE, Embase, Cochrane, Scopus, CINAHL, Clinical key, Google Scholar, and Ovid with selected keywords and explored the reference list of articles. Meta-analysis was performed using the RevMan software version 5.4; p-value < 0.05 was considered statistically significant. The effect of variables was measured in odds ratio (OR) with a fixed-effects model. Six published interventional studies were selected as per the eligibility and participants, intervention, comparison, and outcome (PICO) framed for systematic review and meta-analysis. The search was restricted to articles in the English language, articles published online, and preprinted articles until September 2020. Outcome variables include antenatal and postnatal care utilization by pregnant and delivered mothers. The results have been presented in the form of a forest plot. The findings of this meta-analysis depicted the significant increase in four or more antenatal care (ANC) attendance (OR = 1.81, 95% confidence interval (CI) = 1.49-2.19), tetanus toxoid (TT) immunization (OR = 1.63, 95% CI = 1.17-2.27), compliance to iron supplementation (OR = 1.88, 95% CI = 1.18-3.00), and postnatal care attendance (OR = 2.54, 95% CI = 2.15-2.99) among those pregnant mothers who received mHealth intervention compared with the control group. This meta-analysis creates evidence for the effectiveness of mHealth with pooled data of interventional studies with limited sample sizes. Technology is changing, but even with limited support such as short messaging service (SMS), there was an improvement in antenatal and postnatal service utilization. This meta-analysis concluded that mHealth has the potential to increase the utilization of antenatal and postnatal care compared to standard care, although the level of evidence is moderate.

## Introduction and background

Pregnant women have the right to attain antenatal care (ANC) throughout the pregnancy for the well-being of the fetus and themselves to promote a positive pregnancy outcome [1]. Antenatal care is a term used to deliver appropriate antenatal care to pregnant mothers, inclusive of four or more antenatal checkups and at least one tetanus toxoid (TT) injection with consumption of a minimum of 100 iron-folic acid tablets [2]. A proper antenatal and postnatal checkup provides necessary care to the mother and baby, which helps in timely identification, management, and referral to appropriate facilities to promote a healthy baby’s delivery to a healthy mother to reduce the burden of maternal and neonatal mortality [3]. According to the WHO, nearly 808 women died every day due to preventable causes of pregnancy and childbirth, such as hemorrhage, hypertension, infections, and indirect causes, due to the interaction between preexisting medical conditions and pregnancy, and 94% of them are from low- and middle-income countries (LMICs) [4]. A systematic analysis done by the UN Maternal Mortality Estimation Inter-Agency Group, based on maternal mortality ratio (MMR) sustainable development goal, estimated the global projection of MMR for 2030 to be nearly 161 deaths per 100,000 live births. It is estimated as 357 deaths per 100,000 live births in Sub-Saharan Africa, 115 deaths per 100,000 live births in Southern Asia, 72 deaths per 100,000 live births in Southeastern Asia, and 43 deaths per 100,000 live births in Northern Africa [5]. It is also observed that a woman in low-income countries has 120 times higher risk of mortality due to pregnancy- and childbirth-related causes compared to higher-income countries [4,6]. The probable reasons for such bad pregnancy-related outcomes could be administrative, logistic, and technical failures, along with insufficient financial support and skilled health personnel, poor accessibility, and nonadherence to maternal and child health services [7]. The emergence of mobile technology in health facilitates the opportunity to reach the concerned individual and modify their attitude and behavior for a specific health issue [8]. It can also be an effective tool in sensitizing the population and imparting prenatal education as an engagement tool [9]. Mobile technologies have effectively created a place through robust communication engagement in treating the disease condition that requires treatment adherence and follow-ups [10]. It also has the potential to improve perinatal health outcomes by enhancing the acceptability and accessibility of existing maternal and child health services [11]. We have reviewed relevant studies that have evaluated mobile technology’s effectiveness in promoting maternal and child health by measuring maternal satisfaction, their attitude and behavior toward available maternal and child health services, their antenatal and postnatal attendance, and maternal and perinatal health as well [12-17]. However, it remains inconclusive due to the limited sample size; hence, it is required to integrate and analyze these studies’ findings with a systematic review of all available relevant literature and meta-analysis. This study evaluated mobile health (mHealth) interventions’ effectiveness in antenatal and postnatal care utilization in low- and middle-income countries.

This article has been posted as a preprint on medRxiv (https://medrxiv.org/cgi/content/short/2020.12.22.20248713v1).

## Review

Methods

Data Sources and Search Strategy

We followed the Preferred Reporting Items for Systematic Reviews and Meta-Analyses (PRISMA) 2020 guidelines for the systematic review [18]. We reviewed literature from a broad range of databases - PubMed, MEDLINE, Embase, Cochrane, Scopus, CINAHL, Clinical key, Google Scholar, and Ovid. The search was restricted to articles in the English language, articles published online, and preprinted articles until September 2020. The following keywords were used: “pregnant women,” “antenatal care,” “antenatal attendance,” “ANC visit,” “pregnancy,” “maternal health,” “maternal health services,” “maternal health access,” “pregnant,” “prenatal care,” “ANC Checkups,” “antenatal mothers,” “PNC,” “postnatal,” “postnatal attendance,” “postnatal mothers,” “postnatal care,” “postnatal checkup,” “postnatal visit,” “tetanus toxoid,” “TT,” “iron supplementation,” and “compliance to iron therapy”; “mHealth,” “mobile health,” “mobile intervention,” “text message,” “telemedicine,” “mobile technology,” and “SMS”; and “LMICs,” “resource-limited countries,” and “low- or middle-income country.”

PICO Framework for Eligibility

Participants: The inclusion criteria were pregnant mothers aged 18-49 years in antenatal, intranatal, and postnatal periods and attended ANC visit in institutional or community settings (primary health centers, community health centers, and subcenters). Adolescent females were excluded.

Intervention: Any intervention designed with a mobile phone or smartphone (mHealth) to support pregnant women and their neonates’ health was used. It includes text messages, phone calls, and reminders through the application to increase antenatal care of pregnant and postnatal mothers. We excluded the studies that used mobile technology for any other communication purpose or specific disease.

Comparison: In the control group, we included conventional or routine services or nonexposure to intervention.

Outcomes: Antenatal care utilization includes four antenatal checkups, iron-folic acid supplementation, and two tetanus toxoid immunization. Postnatal care utilization includes postnatal checkups of delivered mothers.

Setting

Inclusion: Studies conducted in a population of low- and middle-income countries were included.

Exclusion: Studies conducted in high-income countries were excluded.

Time Frame

We included studies conducted from 2008 to 2020.

Study Designs

The studies were randomized controlled trials, cluster randomized controlled trials, quasi-randomized trials, case-control studies, and cohort studies. Cross-sectional studies, case studies, and case series were excluded.

Data Extraction

After systematic searching of literature, titles and abstracts were screened by three reviewers (PY, SK, and RK) independently according to the inclusion criteria with the help of Rayyan, a free web‑based software. The remaining two authors (SB and MK) have resolved any discrepancies related to the eligibility of the studies. After screening abstracts and titles, eligible full-text articles were further screened for eligibility, extracted into a data extraction file, and imported to the RevMan software for meta-analysis. Only six articles were included for the final meta-analysis. After analysis, data have been presented in the form of forest plots separately for each outcome.

Evaluation for the Risk of Bias

Three authors (PY, SB, and RK) have evaluated the risk of bias, and any discrepancy was resolved through discussion with other authors (SK and MK). The risk of bias has been evaluated through the Cochrane Risk of Bias Assessment Tool. It is used to assess random sequence generation (selection bias), allocation concealment (selection bias), blinding of participants and personnel (performance bias), blinding of outcome assessment (detection bias), incomplete outcome data (attrition bias), selective reporting (reporting bias), and other bias through the Review Manager software version 5.4 and have been displayed along with forest plot [19]. Relevant file has been imported from the RevMan software to the “Summary of Findings” table GRADE Profiler to create a “Summary of Findings” table [20]. A summary of the intervention effect and a measure of the quality of evidence were noted in the table. Any disagreement was resolved by other reviewers also.

Data Analysis

Meta-analysis was performed using the RevMan software version 5.4 using a Mantel-Haenszel fixed-effects model. P-value < 0.05 was considered statistically significant. The effect of variables was measured in odds ratio (OR) between two groups for all outcomes in the included studies. Statistical heterogeneity across studies was assessed using the I2 test, and values less than 40% were considered to be indicative of might not be significant heterogeneity [21]. Subgroup analysis could be done for four ANC only if statistical heterogeneity is higher than 40%. The cluster randomized controlled trial’s design effect has been calculated to calculate the effective sample size and, accordingly, events in both treatment and control groups [22]. Funnel plots have been created to show an effect estimate against its standard error for each outcome.

Results

**Figure 1:**
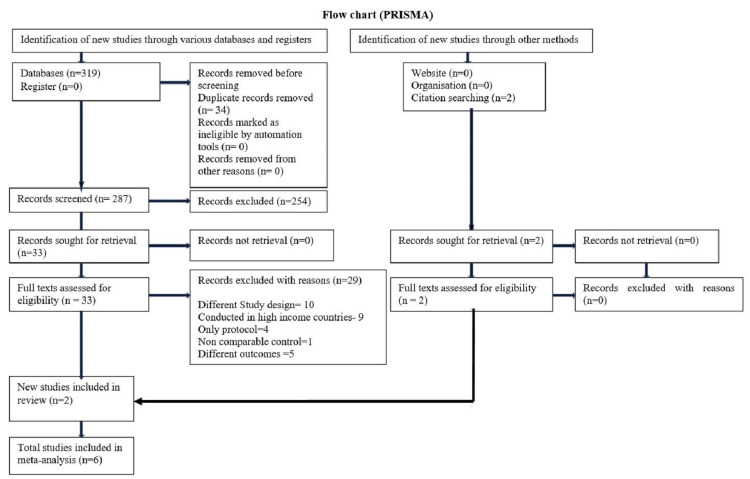
PRISMA flowchart

A total of 321 articles were identified after searching through various databases. The reference lists of the selected articles were also explored to find relevant articles. Further, 287 articles were screened after removing duplicate articles. Thirty-two full-text articles were assessed for eligibility, and 29 articles were excluded because of different study designs, being conducted in high-income countries, different outcomes, noncomparable control groups, and only protocol availability. Only six articles were included for the final meta-analysis (Figure 1).

The total participants for the analysis of four or more ANC outcomes (1115 in the intervention group and 1133 in the control group), TT immunization outcome (601 in the intervention group and 611 in the control group), compliance to iron therapy outcome (369 in the intervention group and 412 in the control group), and postnatal attendance outcome (1499 in the intervention group and 1340 in the control group) were included. The characteristics of the included studies are shown in Table 1.

**Table 1:** Study characteristics SMS: short messaging service

Study	Participants	Setting	Intervention	Comparator	Outcome
Mudey, 2015 [12]	178 interventions and 206 controls	India	SMS reminders	Standard care	Four or more ANC and compliance to iron therapy
Fedha, 2014 [13]	191 interventions and 206 controls	Kenya	SMS reminders	Standard care	Four or more ANC and compliance to iron therapy
Lund, 2014 [14]	1311 interventions and 1239 controls	Zanzibar	An automated SMS system and a mobile phone voucher system	Standard care	Four or more ANC and TT immunization
Kebede, 2019 [15]	173 interventions and 169 controls	Ethiopia	SMS reminders	Standard care	Four or more ANC and postnatal care
Bangal, 2017 [16]	200 interventions and 200 controls	India	SMS reminders	Standard care	Four or more ANC, TT immunization, and postnatal care
Adanikin, 2014 [17]	1126 interventions and 971 controls	Nigeria	SMS reminders	Standard care	Postnatal care

Mudey (2015) [12], Fedha (2014) [13], Kebede (2019) [15], Bangal (2017) [16], and Adanikin (2014) [17] included SMS reminders as an intervention, and Lund (2014) [14] included an automated short messaging service (SMS) system and a mobile phone voucher system as an intervention. All studies had standard care as comparators. Lund (2014) [14] and Kebede (2019) [15] were the cluster randomized controlled trials, and the sample size has been recalculated for the actual sample size considering the study’s design effect. We have performed sensitivity analysis and excluded studies due to methodological issues and different target populations [23,24]. The summary of the findings is shown in Table 2.

**Table 2:** GRADEpro summary of findings ^a^ heterogeneity, ^b^ limited studies, ^c^ wide confidence interval OR: odds ratio; CI: confidence interval

Outcomes	Anticipated absolute effects (95% CI)		Relative effect (95% CI)	№ of participants (studies)	Certainty of the evidence (grade)
	Events with control group	Events with mHealth intervention			
Four or more antenatal checkups	539 per 1000	683 per 1000 (626–734)	OR = 1.84 (1.43–2.36)	2248 (five RCTs)	⨁⨁⨁◯ moderate ^a^
TT immunization	805 per 1000	870 per 1000 (827–903)	OR = 1.62 (1.16–2.26)	1212 (three RCTs)	⨁⨁⨁◯ moderate ^b^
Compliance with iron supplementation	857 per 1000	918 per 1000 (876–947)	OR = 1.88 (1.18–2.99)	781 (two RCTs)	⨁⨁◯◯ low ^b, c^
Postnatal care utilization	583 per 1000	770 per 1000 (722–811)	OR = 2.39 (1.86–3.07)	2839 (three RCTs)	⨁⨁◯◯ low ^b, c^

Table 2 displays the relative effect (95% CI) in odds ratio with events per 1000 participants in both arms. The certainty of evidence and grade are shown as high, moderate, and low for each outcome variable.

**Figure 2:**
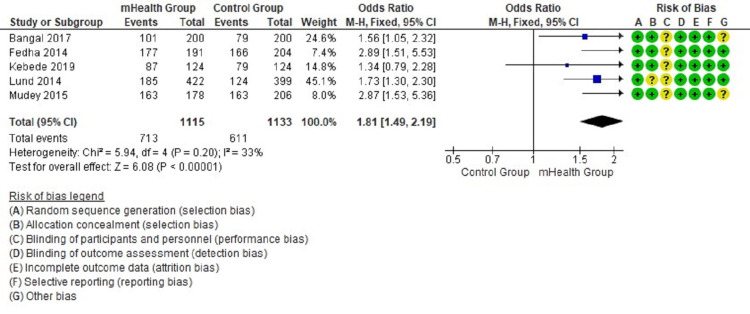
Comparison of four or more antenatal attendance in the intervention and control groups [12-16]

**Figure 3:**
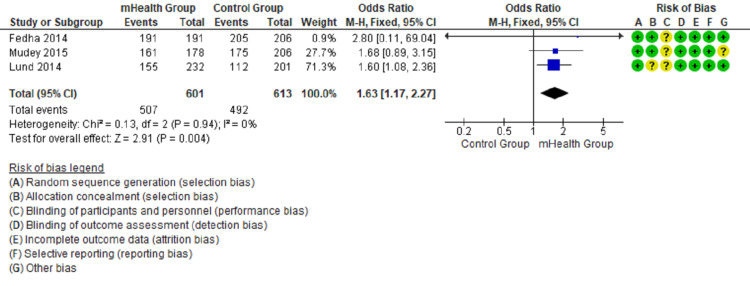
Comparison of TT immunization of pregnant mothers in the intervention and control groups [12-14]

**Figure 4:**
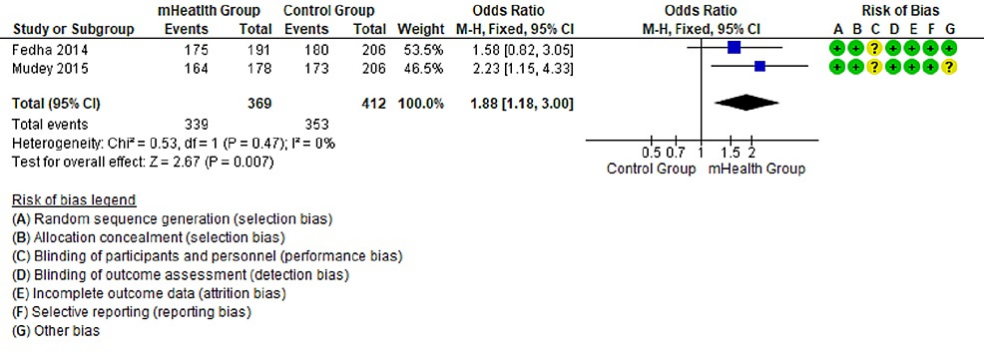
Comparison of compliance with iron supplementation in the intervention and control groups [12,13]

**Figure 5:**
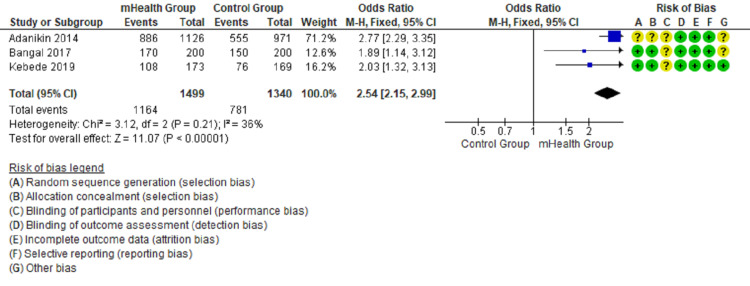
Comparison of postnatal attendance of delivered mothers in the intervention and control groups [15-17]

Forest plots show the comparison between mHealth intervention and the control group with pooled data and favor mHealth intervention’s effectiveness in utilizing antenatal and postnatal care (Figures 2-5).

**Figure 6:**
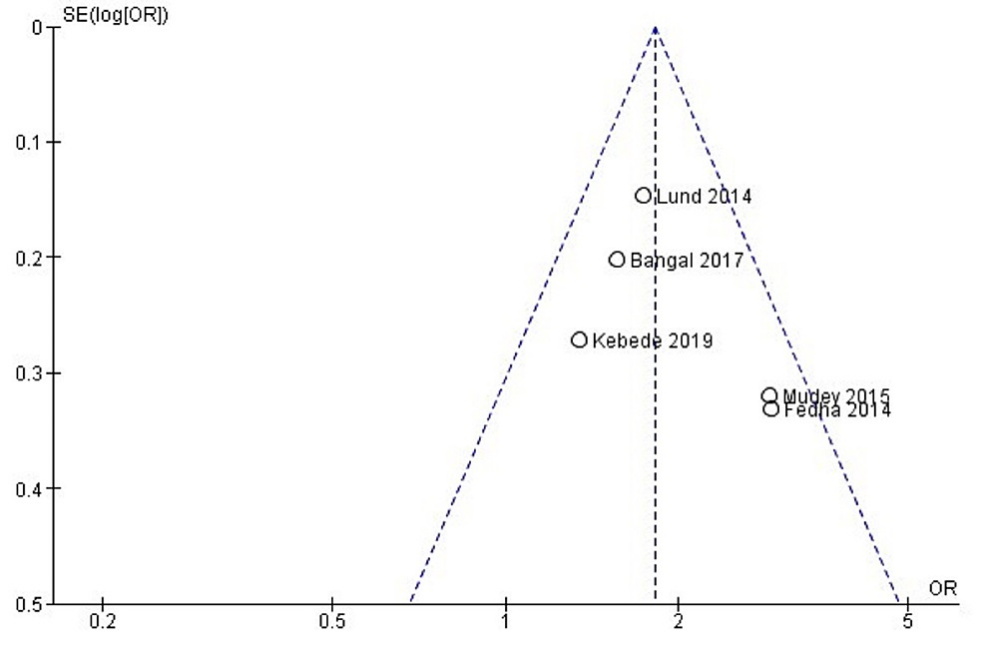
Funnel plot [12-17]

The forest plot in Figure 2 favors the mHealth intervention group (OR = 1.81, 95% CI = 1.49-2.19, I2 = 33%) for the outcome of four or more antenatal attendance. The forest plot in Figure 3 favors the mHealth intervention group (OR = 1.63, 95% CI = 1.17-2.27, I2 = 0%) for the outcome TT immunization of pregnant mothers. The forest plot in Figure 4 favors the mHealth intervention group (OR = 1.88, 95% CI = 1.18-3.00, I2 = 0%) for the outcome of compliance with iron supplementation. The forest plot in Figure 5 favors the mHealth intervention group (OR = 2.54, 95% CI = 2.15-2.99, I2 = 36%) for the outcome of postnatal attendance of delivered mothers. A funnel plot has been created to evaluate publication bias and show an effect estimate against its standard error for antenatal care utilization outcomes (Figure 6).

Discussion

Antenatal care is a term used to deliver appropriate antenatal care to pregnant mothers, inclusive of four or more antenatal checkups and at least one tetanus toxoid injection with consumption of a minimum of 100 iron-folic acid tablets [2], which helps promote the delivery of a healthy baby to a healthy mother [3]. Unfortunately, it was observed that a woman in low-income countries has 120 times higher risk of mortality due to pregnancy- and childbirth-related causes compared to higher-income countries [4,6]. Studies have shown the effectiveness of mHealth intervention toward maternal and child health outcomes [12-17]. In the present meta-analysis, we have pooled the data of the selected studies in low- and middle-income countries to create or strengthen the evidence for mHealth’s effectiveness toward antenatal and postnatal care utilization. This meta-analysis observed that mHealth has the potential to increase antenatal and postnatal attendance compared to the standard approach, although the level of evidence was moderate. The results of this meta-analysis depicted the significant increase in four or more antenatal care attendance, TT immunization, compliance to iron supplementation, and postnatal care attendance among those pregnant mothers who received mHealth intervention in comparison with those who did not receive such intervention. These findings were supported by two systematic reviews performed in low- and middle-income countries, although with low and moderate evidence levels [25,26]. One systematic review suggests that mHealth intervention can be more effective in enhancing maternal and child health services if we target the mothers during antenatal and postnatal periods [26]. If we incorporate the findings of a meta-analysis conducted in China, it also clarifies that mHealth applications with social media can also enhance maternal health [27]. One of the pieces of evidence from the systematic review strengthens the findings that mHealth nutrition interventions also help in improving the dietary intake and nutritional status of pregnant mothers [28]. However, a mixed-method study performed in South Africa shows that mHealth intervention was not useful in improving pregnant mothers’ health knowledge but useful in motivating their self-reported behavior to seek medical services [29].

In the rural population, a community-based randomized controlled trial in Ethiopia supports the positive contribution of SMS-based intervention toward maternal health. We even explored this study but excluded sensitivity analysis due to methodological issues and target populations as community health workers and health extension workers [23]. A randomized controlled trial shows a higher satisfaction level of pregnant women who received SMS during their antenatal duration than the routine antenatal care group. This study also presented the lower anxiety level among pregnant mothers during their antenatal period, but they did not notice any difference in pregnancy outcomes [30]. A controlled quasi-experimental study in Tanzania on pregnant women in improving pregnant mothers’ knowledge on danger signs and birth preparedness also found a significant difference between mHealth and standard care [31]. Ultimately, it is one step toward improving antenatal and postnatal care attendance. As we have discussed, systematic reviews and meta-analyses have been conducted to examine mobile intervention’s effectiveness on different maternal and child health outcomes. However, our meta-analysis findings contribute to mHealth intervention’s effectiveness in utilizing antenatal and postnatal care among pregnant mothers.

Strengths and Limitations of the Study

This meta-analysis creates evidence for the effectiveness of mHealth with pooled data of interventional studies with limited sample sizes. This meta-analysis adheres with the Preferred Reporting Items for Systematic Reviews and Meta-Analyses for Protocols 2009 to ensure the quality of reporting the results. Sensitivity analysis identified the possible reasons for heterogeneity among studies. As we included studies from LMICs, the results can be generalized for the respective population. mHealth as an intervention is a broad term that also created heterogeneity.

## Conclusions

This meta-analysis concluded that mHealth intervention has the potential to increase the utilization of antenatal and postnatal care compared to the standard approach, although the level of evidence was moderate. Technology is changing, but even with limited support such as SMS, there was an improvement in antenatal and postnatal service utilization. It might have a possible solution to enhance maternal and child health services to pregnant mothers and reduce maternal and neonatal mortality. Further studies are required with experimental trials or cluster randomized controlled trials to assess the feasibility and cost-effectiveness of mHealth intervention in community settings with the next step by involving the government health system to implement these findings.
